# Blue‐Ribbon Boys: factors associated with PrEP use, ART use and undetectable viral load among gay app users across six regions of the world

**DOI:** 10.1002/jia2.25130

**Published:** 2018-07-22

**Authors:** George Ayala, Glenn‐Milo Santos, Sonya Arreola, Alex Garner, Keletso Makofane, Sean Howell

**Affiliations:** ^1^ The Global Forum on MSM & HIV (MSMGF) Oakland CA USA; ^2^ University of California, San Francisco San Francisco CA USA; ^3^ San Francisco Department of Public Health San Francisco CA USA; ^4^ Hornet San Francisco CA USA; ^5^ Harvard University Boston MA USA

**Keywords:** HIV, gay men, men who have sex with men, sexual health, HIV services, ART, HIV viral load, gay social network apps

## Abstract

**Introduction:**

Gay social networking apps have grown in popularity among men who have sex with men offering opportunities for rapid and confidential collection of vital data as well as social connection. The goal of our study was to explore factors associated with utilization of pre‐exposure prophylaxis (PrEP) and antiretroviral treatment (ART), and self‐reported undetectable viral load (UVL) using data collected by the gay social networking app Hornet.

**Methods:**

In 2016, the Global Forum on MSM & HIV (MSMGF) partnered with Hornet, to support an educational initiative called Blue‐Ribbon Boys. One aspect of the initiative prompts Hornet users to answer a short series of yes‐no questions about their sexual health. Using survey responses, we evaluated factors associated with PrEP and ART use as well as self‐reported UVL by fitting separate multivariable generalized estimating equation models.

**Results:**

In total, 16,008 unique Hornet users started the survey, of which 12,126 (76%) provided sufficient data for analyses. Of the 10,774 HIV‐negative men, 13% reported PrEP use in the past year. PrEP use was associated with a recent sexually transmitted infection (STI) test or treatment (aOR = 2.19, CI = 1.49 to 3.21); and taking steps to protect oneself from HIV (aOR = 1.41, CI = 1.13 to 1.76). Among HIV‐positive Hornet users (n = 1243), ART use was associated with older age (each year increase aOR = 1.02, CI = 1.01 to 1.04), a recent STI test or treatment (aOR = 4.54, CI = 2.65 to 7.78); and awareness of unlikely HIV transmission with UVL (aOR = 1.53, CI = 1.03 to 2.26). UVL was associated with older age (each year increase aOR = 1.03, CI = 1.01 to 1.04), a recent STI test or treatment (aOR = 4.84, CI = 2.74 to 8.55), and awareness of unlikely HIV transmission with UVL (aOR = 1.98, CI = 1.37 to 2.85).

**Conclusions:**

Study findings underscore the importance of STI testing and treatment as well as information about HIV transmissibility for encouraging PrEP and ART use. Our findings also reveal age disparities, which can undermine incidence reduction among gay men. Gay social networking apps can be effectively used for rapid data collection and sexual health promotion with men who have sex with men. STI testing and treatment programmes offer important opportunities for encouraging PrEP and ART use. Information about HIV transmissibility with consistent ART use should be incorporated into prevention messaging tailored to various age groups.

## 1 Introduction

HIV prevalence among gay and bisexual men and other men who have sex with men is well over 10% worldwide, higher than general population prevalence in many countries 1. In low‐ and middle‐income countries, men who have sex with men are 19 times more likely to be living with HIV compared with people in the general population and represent more than 12% of all new infections each year 2. Although decreases in HIV incidence are well documented, prevalence and incidence are consistently higher among men who have sex with men when compared with other groups 3, 4, 5, 6.

Homophobia can limit the provision and uptake of evidence‐informed and rights‐based HIV prevention, treatment and care services 7, 8, 9, 10. Criminalization of homosexuality encourages human rights abuses, violence, discrimination and stigma, which contribute to health disparities for men who have sex with men 11, 12, 13. Social connectedness with the gay community has been shown to have a protective effect against the often‐devastating effects of homophobia 14, 15, 16, 17, 18. In policy environments that are hostile to lesbian, gay, bisexual and transgender people, men who have sex with men must find creative and discrete ways to find each other for support and health information.

It is therefore not surprising that the use of gay social networking applications has grown in popularity among men who have sex with men, offering opportunities for social and sexual connection. The internet provides benefits to gay and bisexual men who are marginalized or otherwise excluded from mainstream society by providing a safe space and by alleviating social isolation that may result from societal homophobia 19, 20, 21. Social networking applications are an innovative tool for safely reaching marginalized groups like men who have sex with men, especially with expanding accessibility of smart phone technology. They also provide a rapid and confidential means for collecting vital data about the HIV services cascade in hostile country contexts.

In 2016, the Global Forum on MSM & HIV (MSMGF) entered into an innovative public‐private partnership with the geospatial gay social networking application Hornet, to create and launch Blue‐Ribbon Boys. Blue‐Ribbon Boys is a global initiative to educate, empower, and mobilize gay and bisexual men around sexual health. The initiative makes information about HIV pre‐exposure prophylaxis (PrEP), testing, and treatment easily available to both HIV‐negative and HIV‐positive Hornet users in select markets. With 9 million users worldwide at the time it launched Blue‐Ribbon Boys, Hornet was the leading gay social networking app in Brazil, Egypt, Mexico, the Philippines, Russia, Taiwan, Thailand and Turkey. At present, Hornet has 25 million users. The overall goal of Blue‐Ribbon Boys was to create robust demand for unobstructed access to quality sexual health services.

The goal of our study was to explore factors associated with uptake of HIV services among men who have sex with men using data collected through Hornet's Blue‐Ribbon Boys initiative. Specifically, the study aimed to: (1) describe utilization of anti‐retroviral medications (used prophylactically and as treatment for HIV) in a global sample of Hornet users; and (2) examine factors associated with self‐reported PrEP and treatment use and viral suppression in multivariate analyses. Study findings draw attention to the important and unique role gay social apps play in collecting data, promoting sexual health and addressing barriers to HIV service utilization among men who have sex with men that could lead to improvements along the HIV services cascade. In this paper, we use the terms gay and bisexual men, and men who have sex with men interchangeably, with the understanding that sexual orientation and same‐sex sexual behaviour are intricately and specifically influenced by context, culture, community affiliation, identity, gender expression and emotional connection.

## 2 Methods

### 2.1 Participants and procedures

This study is based on a secondary data analysis using responses from a brief online survey that was part of the Blue‐Ribbon Boys initiative, implemented by the gay social networking app, Hornet. Hornet Data collection occurred from November 2015 to April 2016, during which time Hornet users were invited to participate in a brief questionnaire regarding their sexual health. Hornet users were eligible to participate in the study if they self‐identified as male, were age 18 and over, and gave their consent. All participants received a blue ribbon icon on their profile photo signifying their participation in the initiative.

This project followed a two‐step consent procedure. First, Hornet users were provided the app's privacy policy as part of Hornet's terms of service. Second, Hornet users were informed that participation in the survey portion of the Blue‐Ribbon Boys initiative would be voluntary and that responses would be linked with anonymized personal information contained in their user's account online profiles using a unique participant user ID. Survey takers were able to opt‐out of the survey at any time, with no interruption to their app services and at no consequence to their Hornet membership. Users must have read and endorsed the privacy policy before being able to use the app and receive an invitation to participate in the survey. After endorsement of the privacy policy, Hornet users could choose to make their demographic information publicly available to other users via app filters. The privacy policy explicitly restricts use of personal demographic information provided by Hornet users for the purposes of administering app updates, providing customer services, and making available other initiatives, like surveys. In addition, Hornet users are informed that anonymized personal information could be shared in aggregate.

At the completion of the study, sexual health data collected were merged with an anonymized dataset containing study participant personal demographic and behavioural characteristics (e.g. age, relationship status, language, race/ethnicity, country of residence, preferred sexual position/role) using a unique participant user ID. Study procedures were reviewed by the University of California, San Francisco's Institutional Review Board (IRB# 18‐24991, REF# 217272), which determined that the protocol was exempt under Category 4.

### 2.2 Measures

Blue‐Ribbon Boys utilised a short, yes‐no survey, which began with the question: *Do you know your HIV status?* HIV‐negative participants were then asked: *Are you taking PrEP? Have you asked your healthcare provider if PrEP is right for you? Are you taking steps to protect yourself from HIV? In the past year, have you had an test and/or received treatment for a sexually transmitted infection (STI)?* HIV‐positive respondents were asked: *Are you taking anti‐retroviral treatment daily? Have you achieved or maintained an undetectable viral load? Are you aware that if you are undetectable it's virtually impossible to transmit HIV? In the past year, have you had an STI test and/or treatment?* Men who indicated they did not know their HIV status were asked: *In the past year, have you had an STI test and/or received treatment? Are you taking steps to protect yourself from HIV? Do you plan to get an HIV test in the next three months? Have you researched resources for getting an HIV test?* Blue‐Ribbon Boys was offered as an initiative (including its survey) in 71 countries and in languages spoken by most Hornet users across participants’ country of residence, including English, Portuguese, French, Spanish, Thai and Vietnamese. Hornet's market expansion into Latin America, Southeast Asia and Western Europe influenced language offerings.

### 2.3 Statistical analyses

Our primary outcomes of interest were: (1) PrEP use among HIV‐negative participants; (2) antiretroviral treatment (ART) use among HIV‐positive participants; and (3) undetectable viral load (UVL) among HIV‐positive participants. All outcomes of interest were dichotomized to no and yes. We evaluated demographic and behavioural factors associated with of PrEP use, ART use and UVL by fitting separate multivariable generalized estimating equations (GEE) models with a logit link function for our dichotomous outcomes that used an exchangeable correlation matrix with robust standard errors, accounting for clustering by country (n = 71) using all complete data available. Analyses were limited to observations with available data on the outcomes of interest. For model building, the demographic and behavioural characteristics were informed by hypothesized predictors of our outcomes a priori. Associations were considered statistically significant using an α cut‐off of 0.05. All statistical analyses were conducted using STATA version 13.1 (College Station, TX, USA).

## 3 Results

### 3.1 Participant characteristics

In total, 16,008 unique Hornet users started the survey, of which 12,126 (76%) provided sufficient data for analyses. The median age of participants was 25 (IQR = 21 to 30). The study sample was comprised of individuals who identify as Asian (29%), White (20%), Latino/Hispanic (7%) and Black (5%). Thirty‐four percent of participants did not report their race/ethnicity and 2% indicated “other.” More than half of study participants (56%) indicated they were not in a relationship and another 34% did not answer this question. The most frequent languages used by the participants in their profiles were English (38%), Thai (24%), Portuguese (12%) and Spanish (10%). Other languages comprised 17% of the sample. Regionally, most participants were from Asia (50%), North America (17%), Europe (16%), or South America (14%). Most participants reported being HIV negative (n = 8420, 69%), or unsure of their HIV status (n = 2354, 19%), while ten percent (n = 1243) of participants reported being HIV positive (see Table 1).

**Table 1:** Participant demographic characteristics (n = 12,126)

Characteristic	N	% including missing	% excluding missing
Age (years)			
18 to 24	4498	37.1	44.8
25 to 29	2871	23.7	28.6
30 to 34	1336	11.0	13.3
35 to 88	1343	11.1	13.4
Not reported/missing	2078	17.1	–
Race/ethnicity			
Asian, South Asian	3550	29.3	44.2
Black	216	1.8	2.7
Latino	939	7.8	11.7
Other	907	7.5	11.3
White	2426	20.0	30.1
Not reported/missing	4088	33.7	–
Sex role			
Versatile	2347	19.4	57.2
Bottom	835	6.9	20.4
Top	921	7.6	22.5
Not reported/missing	8023	66.2	–
Relationship status			
Single	6830	56.3	85.8
Not single (relationship/companion)	1129	9.3	14.2
Not reported/missing	4167	34.4	–
HIV‐status			
Negative	8420	69.4	70.1
Positive	1243	10.3	10.3
Not sure	2354	19.4	19.6
Not reported/missing	109	0.9	–
Language			
English	4558	37.6	37.9
Spanish	1160	9.6	9.7
Portuguese	1435	11.8	11.9
Thai	2852	23.5	23.7
Other	2012	16.6	16.7
Not reported/missing	109	0.9	–
Region			
Africa	211	1.7	1.7
Asia	6054	49.9	49.9
Europe	1900	15.7	15.8
North America	2026	16.7	16.7
South America	1715	14.1	14.1
Other	220	1.8	1.8

Among the 8420 HIV‐negative Hornet users, 7920 responded to the question regarding daily PrEP use, of whom 13% reported using PrEP. Among the 1243 HIV‐positive Hornet users who responded to the questions regarding ART (n = 1164) and viral load (n = 1145), 79% reported ART (n = 924) use and 79% (n = 900) reported having UVL. Of the HIV‐positive survey participants who responded to the ART awareness question (n = 1118), 81% reported being aware that an UVL made it virtually impossible to transmit HIV (see Table 2).

**Table 2:** Survey responses by sero‐status

	N (%)	
	Yes	No
All (n = 12,126)		
In the past year, have you had an STI test and/or received treatment?	4698 (43.2)	6186 (56.8)
Are you taking steps to protect yourself from HIV?	8877 (88.7)	1127 (11.3)
HIV‐negative (n = 8420)		
Are you taking PrEP?	1041 (13.1)	6879 (86.9)
Have you asked your healthcare provider if PrEP is right for you?	2264 (29.1)	5515 (70.9)
HIV‐positive (n = 1243)		
Are you taking anti‐retroviral treatment daily?	924 (79.4)	240 (20.9)
Have you achieved or maintained an undetectable viral load?	900 (78.6)	245 (21.4)
Are you aware that if you are undetectable it's virtually impossible to transmit HIV?	904 (80.9)	214 (19.1)
Unknown HIV status (n = 2354)		
Do you plan to get an HIV test in the next three months?	1511 (63.3)	875 (36. 7)
Have you researched resources for getting an HIV test?	1383 (57.0)	1045 (43.0)

STI, sexually transmitted infection; PrEP, pre‐exposure prophylaxis.

### 3.2 Factors associated with PrEP use, ART use and UVL

In GEE models adjusting for clustering by country, PrEP use was positively associated with having recently received an STI test or treatment (aOR = 2.19, CI = 1.49 to 3.21); and taking steps to protect oneself from HIV (aOR = 1.41, CI = 1.13 to 1.76). PrEP use was also less likely to be reported by respondents who had their language application set as Spanish (aOR = 0.52, CI = 0.33 to 0.82) and Thai (aOR = 0.33, CI = 0.27 to 0.40) when compared to English speakers. PrEP use was not associated with sexual position or role preferences.

ART use was positively associated with older age (aOR = 1.02; CI = 1.01 to 1.04), having recently received an STI test or STI treatment (aOR = 4.54, CI = 2.65 to 7.78); being aware that HIV transmission is very unlikely with UVL (aOR = 1.53, CI = 1.03 to 2.26); and having the application language set as Portuguese (aOR = 2.21, CI = 1.60 to 3.06) and Thai (aOR = 1.77, CI = 1.32 to 2.39) *versus* English. ART use was not associated with sexual position or role preferences and relationship status.

UVL was also positively associated with older age (aOR = 1.03, CI = 1.01 to 1.04); being aware that HIV transmission is very unlikely with UVL (aOR = 1.98, CI = 1.37 to 2.85); and having recently gotten an STI screen or STI treatment (aOR = 4.84, CI = 2.74 to 8.55). Compared to English‐speakers, respondents who set their application language to Portuguese (aOR = 2.60, CI = 1.88 to 3.61) or Thai (aOR = 2.94, CI = 2.05 to 4.22) were more likely to report having UVL. Having UVL was not associated with sexual position or role preferences and relationship status. Factors associated with PrEP use, ART use and UVL are presented in Table 3.

**Table 3:** Characteristics associated with PrEP use, ART use and UVL

	PrEP among HIV‐negative participants (n = 8420)			ART among HIV‐positive participants (n = 1019)			UVL among HIV‐positive participants (n = 1023)		
	aOR	95% CI	*p*	aOR	95% CI	*p*	aOR	95% CI	*p*
Age (per year increase)	1.00	0.99 to 1.01	0.90	1.02	1.01 to 1.04	**<0.01**	1.03	1.01 to 1.04	**<0.01**
Relationship status									
Single	*Ref*								
Not single (relationship/companion)	1.13	0.93 to 1.36	0.21	1.13	0.58 to 2.19	0.72	1.18	0.64 to 2.19	0.59
Unknown	1.03	0.93 to 1.15	0.56	1.13	0.78 to 1.63	0.51	0.87	0.64 to 1.17	0.34
Sexual position/role preference									
Versatile	*Ref*								
Bottom	1.18	0.94 to 1.49	0.16	1.28	0.55 to 2.94	0.57	1.27	0.63 to 2.56	0.50
Top	0.83	0.68 to 1.02	0.08	0.56	0.21 to 1.48	0.24	0.60	0.35 to 1.04	0.07
Unknown	1.09	0.91 to 1.30	0.34	0.71	0.47 to 1.07	0.10	0.68	0.45 to 1.02	0.06
Language									
English	*Ref*								
Spanish	0.52	0.33 to 0.82	**0.01**	1.44	0.48 to 4.33	0.52	1.30	0.38 to 4.49	0.67
Portuguese	1.09	0.83 to 1.44	0.54	2.21	1.60 to 3.06	**<0.01**	2.45	1.69 to 3.55	**<0.01**
Thai	0.33	0.27 to 0.40	**<0.01**	1.77	1.32 to 2.39	**<0.01**	1.68	1.20 to 2.36	**<0.01**
Other	1.12	0.79 to 1.59	0.54	1.03	0.60 to 1.76	0.92	1.03	0.60 to 1.79	0.91
Unknown	–	–	–	–	–	–	–	–	–
Yes, in the past year had an STI test and/or treatment	2.19	1.49 to 3.21	**<0.01**	4.54	2.65 to 7.78	**<0.01**	4.84	2.74 to 8.55	**<0.01**
Yes, aware that if you are undetectable it's virtually impossible to transmit HIV	–	–	–	1.53	1.03 to 2.26	**0.04**	1.98	1.37 to 2.85	**<0.01**
Yes, taking steps to protect yourself from HIV	1.41	1.13 to 1.76	**<0.01**	–	–	–	–	–	–

PrEP, pre‐exposure prophylaxis; ART, antiretroviral treatment; UVL, undetectable viral load; STI, sexually transmitted infection; GEE, generalized estimating equation.

GEE models for PrEP use, ART use and UVL fitted for different populations based on available outcomes.

## 4 Discussion

Our study examined factors associated with PrEP use, ART use and UVL in a large, international sample of gay and bisexual men who are the users of the popular gay social networking app Hornet. The sample size and median age of Hornet users offered a unique window into the sexual health of a population that is at elevated risk for STIs, including HIV. Ten percent of study participants reported being HIV‐positive. HIV prevalence among gay and bisexual men reported from other online convenience samples range from 12% to 30% 22, 23, 24. Self‐reported HIV prevalence among study participants is troubling given the young median age of this cohort. HIV incidence among gay and bisexual men under the age of 30 is especially high and of concern 25, 26, 27.

Low PrEP use among study participants is not surprising. Although the PrEP landscape has changed dramatically since 2016 when Hornet surveyed its users through the Blue‐Ribbon Boys initiative, PrEP is still not broadly available, especially in low and middle‐income countries 28. Findings like these could help establish important baseline estimates early in the roll‐out of PrEP programmes against which future progress can be measured. Low PrEP coverage is a missed prevention opportunity for gay and bisexual men who are highly motivated about their sexual health and about PrEP as a prevention option 29. For example, in this study, nearly 90% of respondents (n = 8877) reported taking steps to protect themselves from HIV.

ART utilization and viral suppression rates reported by study participants are higher than previously reported among men who have sex with men in other studies 25, 26, 27. This may be due to increasing awareness among gay and bisexual men about the health and prevention benefits of UVL because of community education efforts (e.g. Undetectable = Untransmissible) 30. It could also reflect selection bias towards gay and bisexual men who have better access to the Internet and who tend to be better educated and linked to HIV‐related services 31.

Language differences we found in multivariate analyses are not surprising given the limited availability of PrEP at the time of the study. For example, there is no national PrEP policy or guidance in Thailand and PrEP availability is limited to pilot studies, with additional limited accessibility among gay and bisexual men with private insurance 32. Similarly, PrEP availability was limited in Latin America between 2015 and 2016. It was not until 2017, that the Brazilian Ministry of Health announced their plans to make PrEP available to individuals at elevated risk for HIV, for which gay communities actively lobbied.

Age differences in ART use and UVL reinforce previously reported age disparities in access to services between younger and older men who have sex with men 33. In this study, Portuguese‐ and Thai‐speaking men who have sex with men reported higher ART use and UVL than English‐speaking men, which was an unexpected finding that warrants further investigation. While rolling out the Blue‐Ribbon Boys initiative, Hornet focused its earlier community educational campaigns in countries with their largest market footprint. Those countries included Thailand, Brazil and Mexico. These campaigns instigated robust community mobilization around viral suppression and information about the prevention potential of PrEP and ART. Community efforts in Hornet markets may have influenced findings reported here.

Study participants who reported having had an STI test and/or treatment and reported awareness about the link between viral load and HIV transmissibility were significantly more likely to report ART use and UVL. These findings highlight the critical role sexual health services can play in facilitating access to HIV‐related education and services. Moreover, awareness about risk minimalization may play key role in PrEP and ART uptake 34, which may, in turn, consequently influence viral suppression.

### 4.1 Study strengths and limitations

Our study had several strengths and limitations that are important to note. The study sample of Hornet users was conveniently recruited, restricting generalization of study findings. Online convenience samples can create a selection bias towards gay and bisexual men who have better access to the Internet 31. It should also be noted that survey participants who volunteered, reflect Hornet users who are highly motivated to act on their sexual health. In addition, the cross‐sectional design prevents the identification of causal relationships. The study findings are nevertheless important, given the sample size and the median age of respondents, which was relatively young.

Another limitation from this study is the missing data from the sexual health outcomes of interest. As mentioned, our analysis was restricted to individuals who provided information about these outcomes. It is possible that this approach has introduced a selection bias toward participants who are more comfortable discussing their sexual health and this group may be different from broader Hornet users who were excluded. In addition, due to the anonymized nature of this study, we are unable to compare our participants with Hornet users who declined to participate. Therefore, we do not know if there are significant differences between our sample, and those who declined to participate. Nevertheless, because our survey invitation was extended via the use of the Hornet app, we speculate that individuals with unlimited data plans and/or have reliable wi‐fi access (and potentially have more resources) were more likely to participate in the study. Hence, our findings may likely overestimate self‐reported PrEP or ART use and HIV viral suppression among men who have sex with men.

## 5 Conclusions

Study findings underscore the importance of STI testing and treatment sites as entry points for encouraging PrEP and ART use. Information about HIV transmissibility and viral load should be integrated into tailored HIV prevention messages and educational campaigns. Moreover, sexual health programmes should be designed with age disparities in service access and utilization in mind. Structural‐level factors like national HIV policies may be influencing availability and utilisation of important prevention tools, like PrEP. As illustrated by this study, gay social networking apps offer important opportunities to rapidly collect data and disseminate tailored prevention messages. Online social networking applications should be better studied and utilized to more fully leverage their contribution to sexual health promotion among men who have sex with men 35. Specifically, the HIV sector may benefit from the broad‐based adoption of agile approaches to programme design used by social networking apps like Hornet 36, 37. Such approaches may lead to more user‐friendly programmes, which may encourage better uptake of ART and PrEP use and more finely tailored prevention interventions for men who have sex with men 38.

## Competing interests

Authors have no competing interests to declare.

## Authors’ contributions

GA is the principal investigator of the Blue‐Ribbon Boys study and was the lead writer. GMS is a co‐principal investigator, led all data analysis and co‐wrote the methods and results reported here. SA, AG, KM and SH are co‐ investigators who assisted with the overall design of the study and offered feedback to the draft manuscript.
